# Supplementary material for: The causal relationship between the human gut microbiota and pyogenic arthritis: a Mendelian randomization study
Source: Front Cell Infect Microbiol. 2024 Nov 26;14:1452480. doi: 10.3389/fcimb.2024.1452480 (PMC11629706; doi:10.3389/fcimb.2024.1452480)
Supplement: Supplementary file 1 [file DataSheet1.zip › mendelian test/ebi-a-GCST90027828.csv_leave_one_out.pdf]

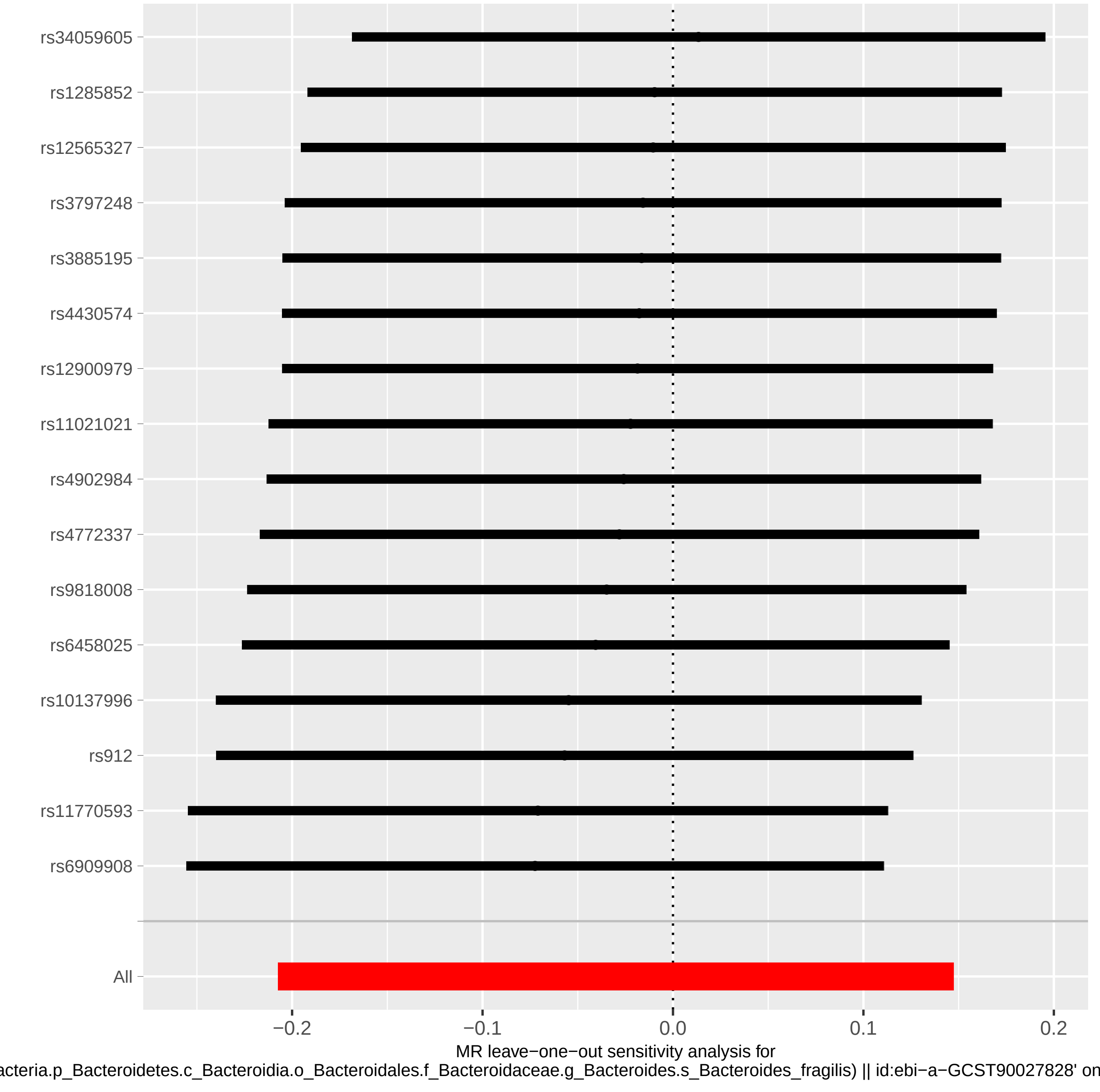
rs34059605
rs1285852
rs12565327
rs3797248
rs3885195
rs4430574
rs12900979
rs11021021
rs4902984
rs4772337
rs9818008
rs6458025
rs10137996
rs912
rs11770593
rs6909908
All
−0.2
−0.1
0.0
0.1
0.2
MR leave−one−out sensitivity analysis for
acteria.p_Bacteroidetes.c_Bacteroidia.o_Bacteroidales.f_Bacteroidaceae.g_Bacteroides.s_Bacteroides_fragilis) || id:ebi−a−GCST90027828' on
